# GPR39 Is Coupled to TMEM16A in Intestinal Fibroblast-Like Cells

**DOI:** 10.1371/journal.pone.0047686

**Published:** 2012-10-25

**Authors:** Fanning Zeng, Nicholas Wind, Conor Mcclenaghan, J. Martin Verkuyl, Robert P. Watson, Mark S. Nash

**Affiliations:** Gastrointestinal Disease Area, Novartis Horsham Research Centre, Horsham, United Kingdom; Loyola University Chicago, Stritch School of Medicine, United States of America

## Abstract

GPR39 is a GPCR implicated as a regulator of gastrointestinal motility, although the mechanism remains elusive. Here, we report that GPR39 is expressed by a specific cell population cultured from mouse small intestine muscle layers, which was subsequently identified as fibroblast-like cells (FLCs) that have recently been shown to modulate gut motility. Application of the GPR39 agonist, Zn^2+^, induced large currents and membrane depolarization in FLCs cultured from wild-type mice, but not *Gpr39* ^−/−^ mice. This Zn^2+^-induced current could be suppressed by application of a TMEM16A antagonist, CaCC_inh_-A01, or by silencing *Tmem16a* expression. These data suggest that GPR39 might modulate gut motility via regulating TMEM16A function in FLCs.

## Introduction

GPR39 is a G protein-coupled receptor (GPCR) found in all vertebrates. Together with *Gpr38*, *Gpr39* was cloned as a novel member of the ghrelin and neurotensin receptor families [1]. While GPR38 has since been deorphanized as the receptor for motilin [2], an important peptide regulator of GI tract motility [3], the natural ligand of GPR39 is still a subject of discussion. In 2005, Zhang *et al.* reported GPR39 to be the receptor for obestatin [4]. However, studies by others have contradicted this finding [5], [6], and demonstrated the inability of obestatin to bind GPR39 *in vitro*. Instead, Zn^2+^ has been proposed as an endogenous agonist for GPR39 [5], [7], [8]: exogenously applied Zn^2+^ stimulates inositol 1,4,5-trisphosphate production, as well as induces Ca^2+^ signals in GPR39-expressing cells; the potency and efficacy of Zn^2+^ on GPR39 suggests that it could well be physiologically relevant modulator of GPR39 *in vivo*.

GPR39 is most highly expressed in the pancreas, gastrointestinal (GI) tract, liver and kidney [9], [10]. Other tissues that express GPR39 include adipose tissue, thyroid, and heart, as well as some regions of the brain [9]. As to its physiological function, GPR39 is involved in insulin release and pancreatic function [5], [11]. It has also been implicated as a regulator of GI motility since *Gpr39* ^−/−^ mice display an acceleration of gastric emptying and a more effective colonic expulsion [10]. However, little is known about the mechanism by which GPR39 regulates gut motility.

Regulation of GI motility takes place through the coordinated activities of enteric and sensory neurons, smooth muscle cells (SMCs), as well as interstitial cells of Cajal (ICC). Endocrine or paracrine factors associated with secretory glands and the gut flora also play a role [12]. More recently, FLCs are suggested as a new class of excitable cells that might also be involved in the control of GI motility [13]. In the gastrointestinal tract, FLCs form a cellular network with their processes and mirror the anatomical distributions of ICC [14]. They can be distinguished from ICC by their robust expression of platelet derived growth factor receptor *α* (PDGFRα) and lack of c-Kit expression. It has been suggested that FLCs help with spreading of the slow waves generated by the ICC [15]; or, because FLCs locate near terminals of enteric motor neurons and form a syncytium with SMCs via gap junctions [13], [14], they might be involved in motor neurotransmission in GI tract.

To increase understanding of the role of GPR39 in the gut, we first conducted extensive work using commercially available and in-house generated GPR39 antibodies to localise expression. However, we were unable to generate robust and convincing data on the cell-types expressing GPR39, and therefore focussed on functionally localising GPR39 in isolated gut cells. In the present study, we report that functional GPR39 is highly expressed by a specific cell population cultured from GI muscle layers, which were subsequently identified as intestinal FLCs. Activation of GPR39 by Zn^2+^ not only resulted in evident Ca^2+^ signals in the cultured FLCs, but also induced large TMEM16A-dependent currents and membrane depolarization. These data suggest that GPR39 is functionally coupled to TMEM16A channels in cultured intestinal FLCs.

**Figure 1:**
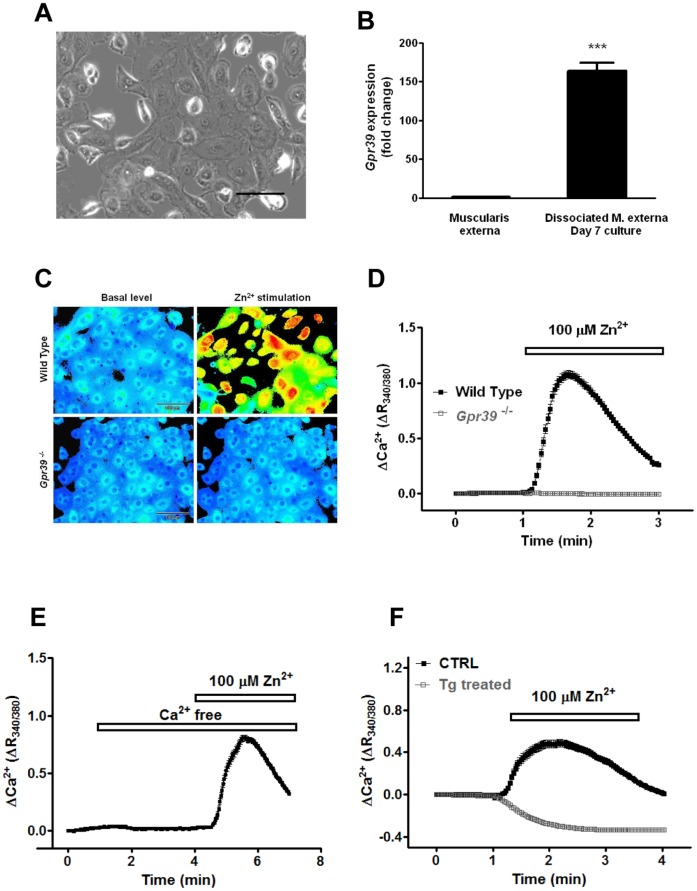
GPR39-expressing cells. (**A**) a bright field image of cobblestone-like cells. Scale bar = 100 µm. (**B**) *Gpr39* mRNA levels were evaluated by Taqman gene expression analysis in both muscle tissues and cultured cells. After 7 days in culture, *Gpr39* levels were up-regulated 163.9 fold in the cultures (n = 3). (**C**) Cells were loaded with Fura2-AM. Shown are ratiometric images of Ca^2+^ signals before and after 100 µM Zn^2+^ challenge. [Ca^2+^]*_i_* is indicated on a rainbow scale with blue representing low [Ca^2+^]*_i_* and orange/red high [Ca^2+^]*_i_*. (**D**) Ca^2+^ signals were elicited in cells cultured from wild-type mice, but not *Gpr39* ^−/−^ mice. (**E**) Zn^2+^ induces Ca^2+^ signals in Ca^2+^ free bath solution. (**F**) Cells were pre-incubated with or without 1 µM thapsigargin to deplete intracellular Ca^2+^ stores. Thapsigargin treatment not only abolished Zn^2+^ elicited Ca^2+^ signals, but also resulted in a decrease in [Ca^2+^]_i_ after Zn^2+^ application, the mechanism of which is still unknown.

## Results

Gastrointestinal (GI) motility is tightly controlled by the enteric nervous system. Therefore, GPR39 might regulate gut motility by influencing the activity of enteric neurons. However, no Zn^2+^-induced Ca^2+^ signals were observed in cultured enteric neurons (Fig. S1). Furthermore, we observed no changes in cAMP or IP3 signaling pathways when the cultures were challenged GPR39 agonist (data not shown), suggesting the expression of functional GPR39 in enteric neurons is low. To further explore the cell type expressing GPR39, we set up a culture using dissociated muscle layers from mouse small intestine. Over the culture period, a highly proliferative cell population gradually dominated the culture, accounting for more than 90% of the cells in the culture. These proliferating cells showed a distinct morphology of cobblestones under the microscope (Fig. 1A). Real time PCR analysis showed that expression of *Gpr39* was up-regulated more than 160-fold in the cultures as a result of the enrichment of this cell-type after 7 days *in vitro* (163.9±10.5 fold, n = 3, *P*<0.0001, Fig. 1B). When challenged with Zn^2+^, a robust but transient Ca^2+^ signal was evident (n = 176, Fig. 1C and D). In contrast, none of those cobblestone-like cells cultured from *Gpr39* ^−/−^ mice showed any response to Zn^2+^ (n = 565).

**Figure 2:**
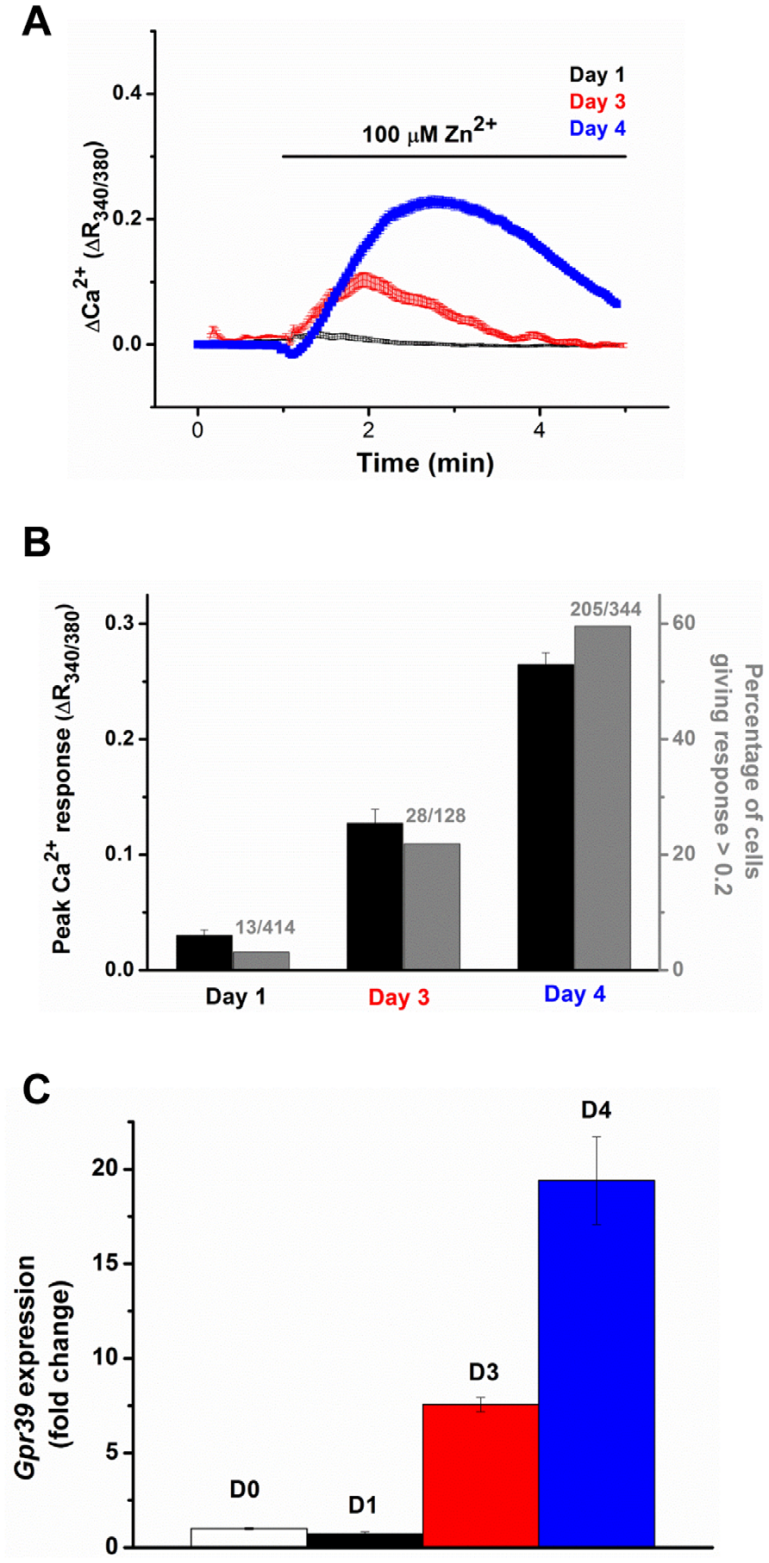
Correlation between GPR39 expression and function in the culture cells. (A) the Zn^2+^ induced Ca^2+^ signals were measured in the cultured cells after 1, 3 and 4 days *in vitro*. (B) a bar chart summarizing the total Ca^2+^ response and percentage of Zn^2+^ responsive cells in Panel A. (C) shows the expression of *Gpr39* mRNA in the corresponding cultures over the same culturing periods.

To illustrate the origin of the Ca^2+^ signals, Ca^2+^ was removed from the external bath solution before challenging the GPR39-expressing cells with Zn^2+^: the [Ca^2+^]_i_ rises remained unaffected (Fig. 1E). In contrast, Ca^2+^ signals were completely attenuated by the pre-treatment with thapsigargin (Tg) to deplete intracellular Ca^2+^ stores (Fig. 1F). Thus, as predicted for such a Gq-coupled GPCR, Zn^2+^-dependent Ca^2+^ responses in the cobblestone-like cells were mediated by release of Ca^2+^ from intracellular stores via GPR39 activation.

**Figure 3:**
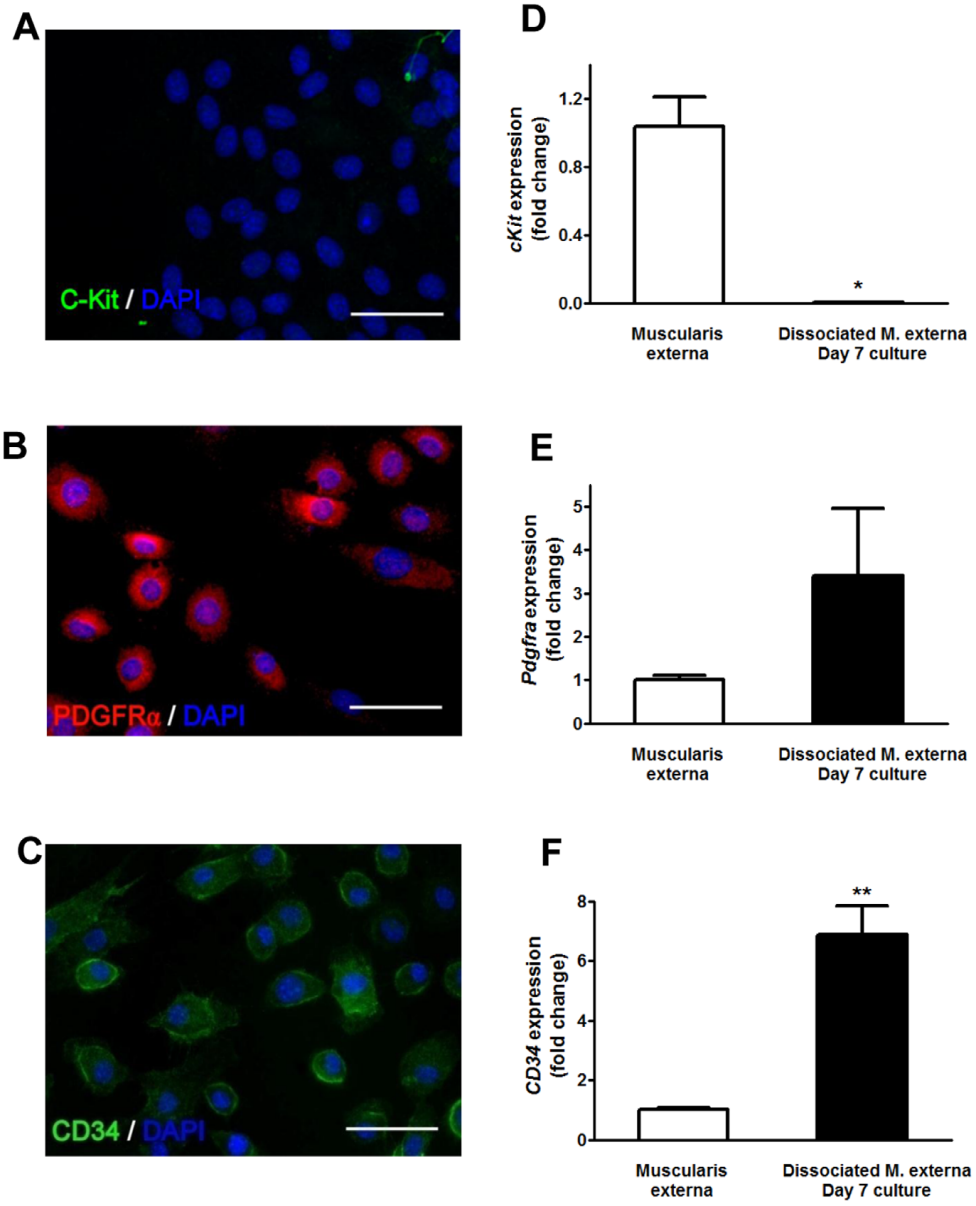
GPR39 expressing cells are fibroblast-like cells. (**A-C**) Representative images for immuno-labelling of C-Kit, CD34, PDGFRα and SK3 in cultured cobblestone-like cells. Scale bar = 50 µm. (D-F) the mRNA expression levels of *CD34* and *pdgfra* in the cultures increased 6.87±0.96 (p<0.01) and 3.39±1.55 (p>0.05) fold respectively, while c-kit expression decreased 0.0013±0.0003 (p<0.05) fold.

Over the culturing period, in parallel to up-regulation of *Gpr39* mRNA expression, both the percentage of Zn^2+^ responding cells and the total Ca^2+^ response are increased in the cultures established from wild-type (WT) mice (Fig. 2), suggesting the domination of these cells in the culture. To further confirm the correlation between the cobblestone morphology and GPR39 function in the primary cultured cells, we analyzed Ca^2+^ responses to Zn^2+^ application in the cultures from wild-type mice: indeed, Zn^2+^ elicited Ca^2+^ signals in all cobblestone-like cells, while cells with other morphologies only responded to ATP (Fig. S2). Together, it is concluded that Zn^2+^ induces Ca^2+^ signals in these cobblestone-like cells via GPR39 activation.

**Figure 4:**
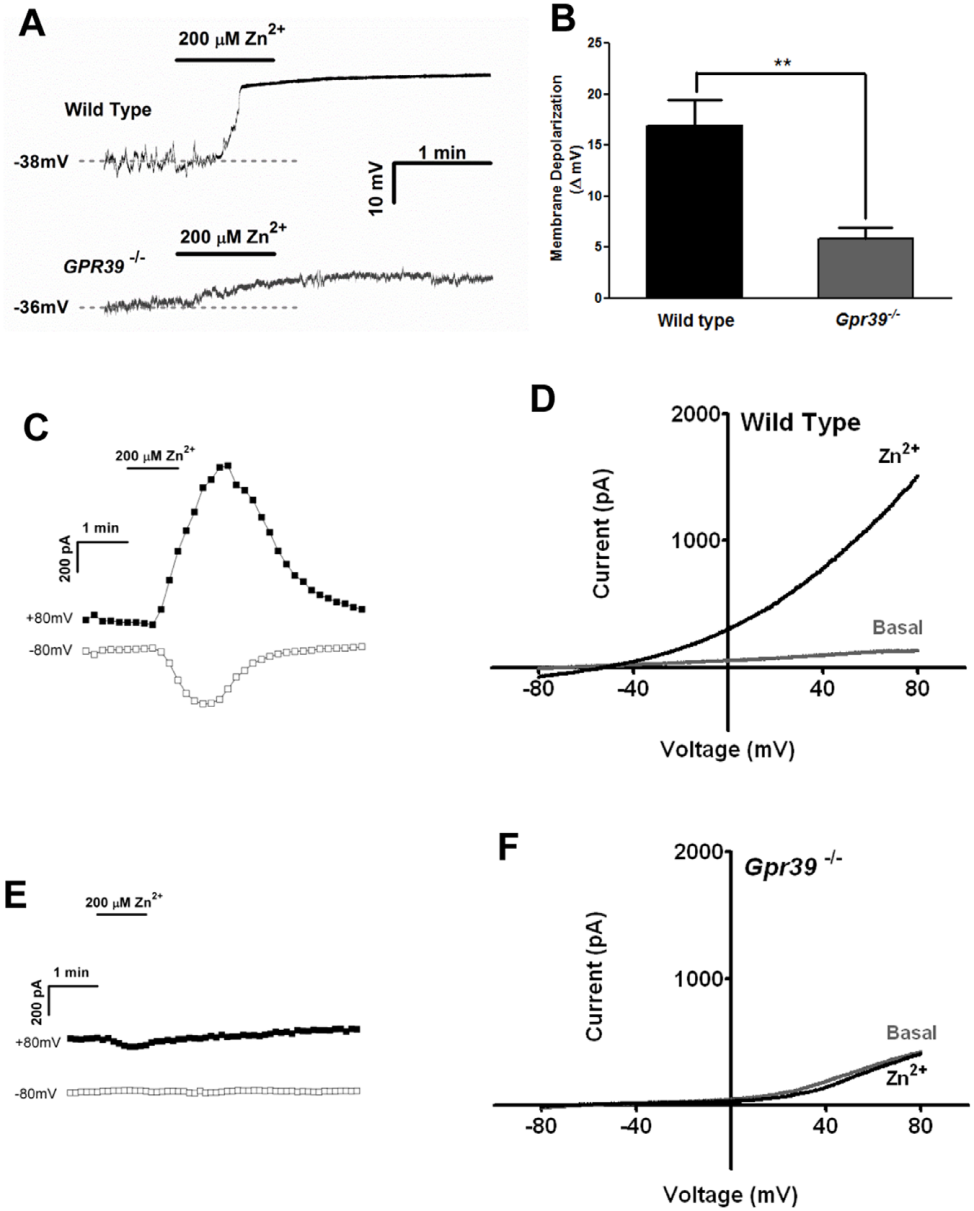
Zn^2+^ induces a large current in FLCs. (**A**) Membrane potentials were recorded in current clamp configuration. Zn^2+^ induced rapid membrane depolarization in FLCs cultured from wild-type mice, but not *Gpr39* ^−/−^ cells. (**B**) Bar chart summarizing Zn^2+^ induced membrane depolarization in FLCs as in panel A. p<0.01. (**C** and **D**) Zn^2+^ induced a large current in wild-type. Current was sampled at +80 and −80 mV. (**C**) shows a time-series plot for a typical Zn^2+^-induced current in FLCs (n = 21). (**D**) shows the corresponding I-V relationship. (**E** and **F**) are similar experiments using FLCs isolated from GPR39 knockout mice and show that no Zn^2+^-induced current was observed in *Gpr39* ^−/−^ FLCs (n = 11).

The muscularis externa of the GI tract contains SMCs, enteric neurons, glial cells, immune cells and several classes of interstitial cells, including ICC and FLCs [13]. In order to determine the identity of the cobblestone-like cells, we performed immunostaining with various cellular markers. The cells were not immunopositive for neuronal (βIII tubulin) or glial (GFAP) markers, the marker for SMCs (smooth muscle α-actin) (data not shown), nor for the ICC marker c-Kit (Fig. 3), but were strongly immunopositive for CD34 and PDGFRα. Similarly, up-regulation of *CD34* and *pdgfra* expression, as well as down-regulation of *c-kit* was observed in these cultures (Fig. 3). Also, these cobblestone-like cells showed positive immunostaining for a small-conductance Ca^2+^-activated K^+^ channel (SK3) (Fig. S3). The expression of CD34, PDGFRα and SK3 has previously been used to differentiate FLCs from other cell types in the gut [13]–[15]. Collectively, these data suggest that the cobblestone-like cells described here are likely to be FLCs, and referred to as such in the rest of the manuscript.

**Figure 5:**
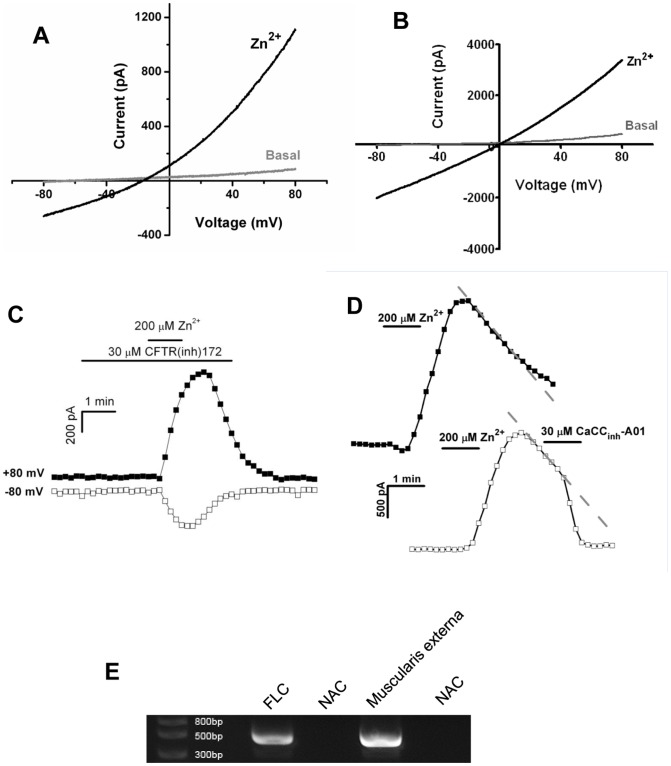
Zn^2+^ induced currents are carried by chloride channels. (**A**) Zn^2+^ induced current when potassium channels were blocked by CsCl in the pipette solution, excluding a major role of K^+^ channels (n = 5). (**B**) Zn^2+^ still induced a current when recordings were conducted in symmetrical NMDG-Cl solutions, suggesting chloride channels are involved (n = 4). (**C**) CFTR antagonist did not have any effect on Zn^2+^-induced currents (n = 3). (**D**) Time courses of the current measured at +80 mV from individual cells. Zn^2+^-induced currents decreased gradually after reaching peak amplitude (top). Application of a TMEM16A antagonist resulted in a rapid blockade of this current (bottom) (n = 7), suggesting TMEM16A is involved. (**E**) Expression of TMEM16A in FLCs was detected by reverse transcription PCR using gene specific primers. NAC stands for no amplification control.

FLCs form an excitable network within muscle layers, and have gap junctions with circular and longitudinal SMCs. Recently Kurahashi *et al.* proposed that FLCs might be involved in motor neurotransmission in GI tract [13]. We therefore examined whether a GPR39 agonist could affect the membrane potential of cultured FLCs. Perforated patch was used to preserve intracellular integrity. To ensure the maximum Ca^2+^ response in FLCs, we challenged the cells with 200 µM Zn^2+^ (EC_50_ = 30 µM in FLCs as measured with Ca^2+^ imaging, Fig. S4). For FLCs cultured from WT or *Gpr39* ^−/−^ mice, no significant difference was observed between their resting membrane potentials (−40±1.48 mV vs −37±1.31 mV, p>0.05, n = 23 and 19). As shown in Figure 4A and 4B, brief exposure to Zn^2+^ led to a rapid depolarization of FLCs (16.9±2.49 mV, n = 5), while no such effect was observed in FLCs cultured from *Gpr39* ^−/−^ mice (n = 6). Next, we investigated whether whole-cell currents were stimulated by Zn^2+^ in FLCs. Shortly after Zn^2+^ application, an outward rectifying current developed that peaked within 2 minutes and rapidly decreased thereafter (Fig. 4B and C, 1736±198.3 pA, n = 21) (again, all FLCs identified by the cobblestone morphology showed responses to Zn^2+^). As expected, no such current was observed in cells cultured from *Gpr39* ^−/−^ mice (Fig. 4D and E, n = 11). These observations demonstrate that activation of GPR39 resulted in stimulation of currents in FLCs.

**Figure 6:**
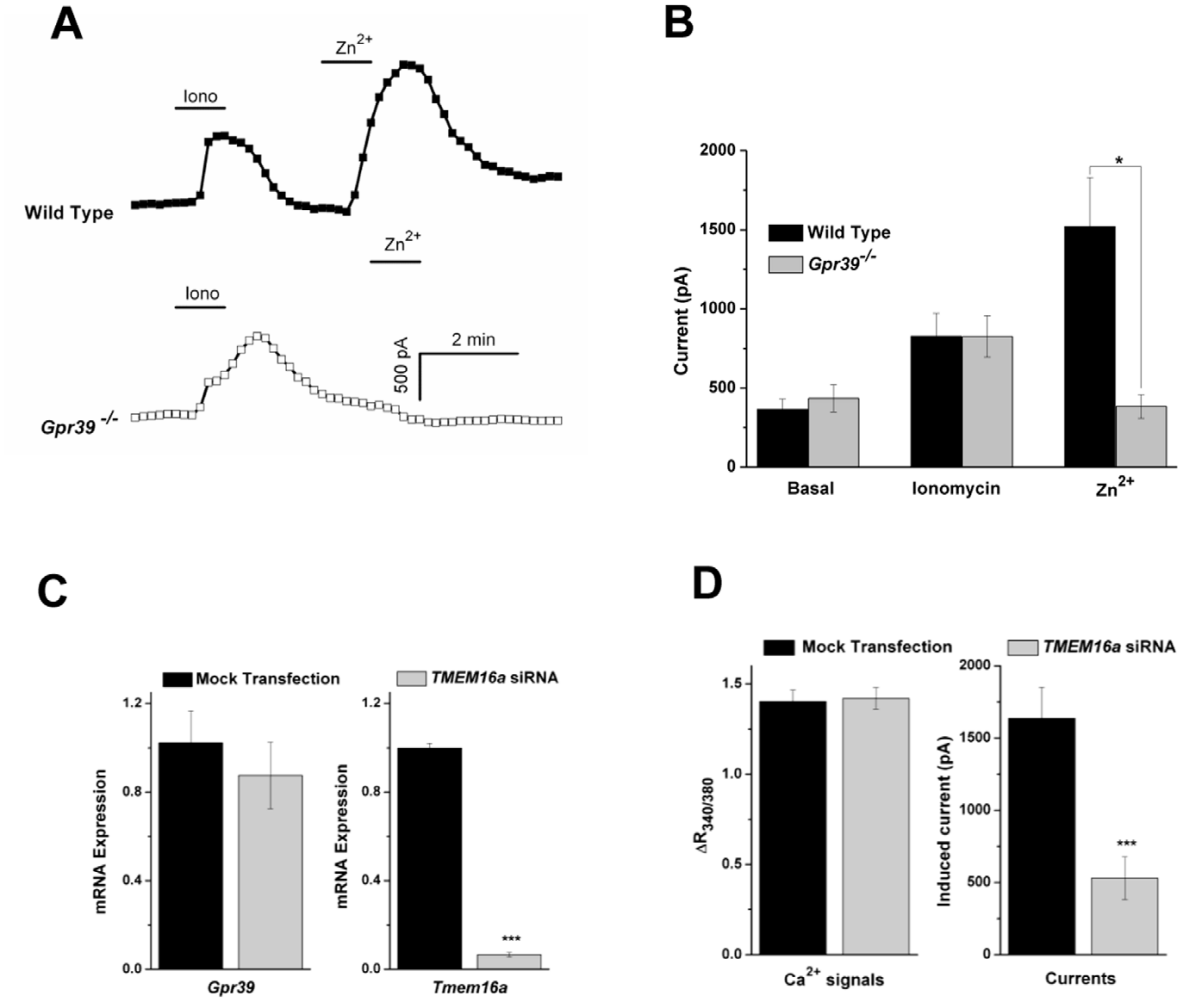
GPR39 activation is functionally linked with TMEM16A channel opening. (**A**) Time courses for the current measured at +80 mV from individual cells. Both Ionomycin (1 µM) and Zn^2+^ evoked currents in FLCs from WT mice (n = 11, top trace), while Zn^2+^ was ineffective in *Gpr39* ^−/−^ FLCs (n = 6, bottom trace). The data are summarized in panel (**B**). (**C**) Knockdown of *Tmem16a* by siRNA in FLCs were verified by Taqman PCR (94% reduction in *Tmem16a* expression), whereas *Gpr39* expression were unaffected (n = 3). (**D**) Zn^2+^-induced Ca^2+^ responses were measured in FLCs. No difference was observed between control and *Tmem16a* siRNA transfected cells (n = 54 and 67). In contrast, the size of Zn^2+^-induced current was reduced to about 32% in transfected cells (n = 7 and 12).

At its peak, the Zn^2+^-induced current reversed at around −40 mV indicating that it is likely to be carried by either potassium or chloride channels. To determine the type of channel involved, we used CsCl in pipette solutions to block potassium channels. Large currents were observed after Zn^2+^ application (Fig. 5A, 1590.5±442.9 pA, n = 5, p>0.05 compared with Fig. 4C), suggesting potassium currents were unlikely to be the major component of the Zn^2+^-induced current in FLC. To further confirm that the Zn^2+^-induced current is a chloride current, whole-cell recordings were performed with symmetrical *N*-methyl-D-glucamine chloride (NMDG-Cl) solutions: both extracellular and pipette solutions contained NMDG-Cl, so that cation currents were minimized and the chloride current was the only channel activity that could be recorded. Upon Zn^2+^ stimulation, a prominent current was observed (Fig. 5B, n = 4). The reversal potential was around 0 mV, which reflects the symmetrical Cl^−^ concentrations across the membranes. Together, these data imply that activation of GPR39 leads to the opening of a chloride channel in FLCs.

The GI tract contains numerous chloride channels, including cystic fibrosis transmembrane regulator (CFTR) [16]. However, the lack of CFTR antagonist activity excluded the involvement of this channel in Zn^2+^-induced currents (Fig. 5C, n = 3). TMEM16A is another chloride channel that has been reported to be highly expressed in the GI tract [17]. To explore whether the Zn^2+^-induced Cl^−^ current was carried by TMEM16A, a TMEM16A antagonist, CaCC_inh_-A01, was tested [18]. It blocked the channel acutely when it was applied after the channel had been activated by Zn^2+^ (the current decrease at the rate of 610.67±107.8 pA/min before, and 2595.85±241.1 pA/min after CaCC_inh_-A01 application, p<0.001) (Fig. 5D, bottom trace, n = 7). Furthermore, when CaCC_inh_-A01 was applied prior to Zn^2+^ administration, no current was observed (1636±214.3 pA in control cells, −81.4±82.1 pA in CaCC_inh_-A01 pre-treated cells, p<0.001, n = 8) (Fig. S5). Thus, CaCC_inh_-A01 effectively blocked the Zn^2+^-induced Cl^−^ current in FLCs, suggesting this current could be carried by TMEM16A channels. Indeed, *Tmem16a* expression was readily detected in cultured FLCs by reverse transcription PCR (Fig. 5E).

As shown in Figure 4, the Zn^2+^ induced Cl^−^ current is absent in *Gpr39* ^−/−^ FLCs. However, if this current is indeed carried by TMEM16A, and given TMEM16A is a Ca^2+^-activated Cl^−^ channel, such a current should be evoked in *Gpr39* ^−/−^ FLCs by raising intracellular [Ca^2+^]. To test this, we examined the effect of ionomycin, a Ca^2+^-specific ionophore, on FLCs. As shown in Figures 6A and 6B, in FLCs cultured from *Gpr39* ^−/−^ mice, Zn^2+^-induced currents are absent, whereas ionomycin-induced currents are similar to those in WT FLCs (826.4±147 pA in WT *vs* 825.5±131 pA in *Gpr39* ^−/−^, p = 0.83, n = 11 and 6). Therefore, as predicted for TMEM16A, Ca^2+^ alone is sufficient to activate this current in FLCs.

To further confirm the identity of the channel, we knocked down *Tmem16a* expression in FLCs by siRNA transfection. Three days after transfection, *Tmem16a* expression was down-regulated by 94% in the transfected cells, while no significant changes in *Gpr39* expression were observed (n = 3, Fig. 6C). Accordingly, GPR39 and TMEM16A activities were also measured in those cells: Zn^2+^-induced Ca^2+^ responses were unaffected by *Tmem16a* silencing (ΔR_340/380_: 1.4±0.06, n = 54, in control; 1.42±0.06, n = 67, in transfected cells, p = 0.83), while Zn^2+^-induced Cl^−^ currents were reduced to 32% in transfected FLCs (1636±214.3 pA in control *vs* 529.5±147.1 pA in transfected cells, p<0.001, n = 7 and 12) (Fig. 6D). Similar results were observed when another *Tmem16a* siRNA was used (data not shown). Collectively, our data suggest that activation of GPR39 is functionally linked to the opening of TMEM16A channels in FLCs.

## Discussion

Despite the evidence that GPR39 might be involved in regulation of GI motility, most GPR39 expression was suggested to be in enterocytes within the epithelium of GI tracts [10]. Expression in enteric neurons was also reported [10], however we did not observe any Zn^2+^ induced responses in cultured myenteric neurons. Thus, it is difficult to explain how GPR39 affects GI motility. Using a culture established from the intestinal muscular layer, we identify a cobblestone-like cell population that expresses functional GPR39. They are likely to be FLCs or PDGFR*α*+ cells as suggested by immunocytochemistry results [13], [14]. Therefore, functional GPR39 expression in intestinal FLCs might account for its role in regulation of GI motility. Indeed, we observed changes of membrane potentials in the cultured FLCs upon GPR39 activation, which is in line with the idea that FLCs might be excitable cells and able to modulate SMC activity via a syncytium [13].

Ca^2+^-activated Cl^−^ channels (CaCC) play a basic or at least a modulatory role in many tissues, including various sensory cells, different types of smooth muscles, heart, endothelium, neuronal tissues, and epithelial organs [19]. They mediate Ca^2+^-dependent Cl^−^ secretion in glands and epithelia, and modify cellular responses to various stimuli in muscle, nerve and receptors. [19]. As a major component of CaCC, the importance of TMEM16A in GI physiology is underscored by the diminished rhythmic contraction in GI tracts of *Tmem16a* knockout mice [17], [20]. Immunochemistry results suggest TMEM16A is expressed by SMCs in various tissues such as the airway, reproductive ducts and oviduct. However, in the GI tract, robust expression of TMEM16A has only been reported in the ICC network [17], [20], which serves as the pacemaker of GI tract contractility. Although these earlier studies did not identify any other TMEM16A expressing cell-type, our data suggest that in addition to ICCs, prominent TMEM16A currents are also present in FLCs.

Activation of TMEM16A can be achieved by application of ionomycin, a Ca^2+^-specific ionophore. It has been also reported that various GPCRs can be functionally coupled with TMEM16A. When over-expressed in HEK293 cell, TMEM16A has been shown to link to M3 or P2Y2 receptors [21], [22]. In small nociceptive neurons from rat dorsal root ganglia, activation of B_2_ receptors by Bradykinin robustly opens TMEM16A [23]. Zhu *et al*. also demonstrated that TMEM16A current in ICCs can be elicited by muscarinic activation [22]. In line with these reports, our results suggest that TMEM16A is functionally linked with GPR39 in the intestinal FLC population, and might serve as a downstream effector of GPR39. Interestingly, we noticed that Zn^2^-induced currents are significantly larger than those induced by ionomycin (Fig. 6A), which suggests that other factors might be involved in TMEM16A activation in these cells.

How GPR39 regulates gut motility remains unclear. However, given the potential role for FLCs in GI function [13] and the importance of TMEM16A in regulation of GI motility [20], it is tempting to speculate that the motility changes observed in *Gpr39* ^−/−^ mice are the result of disrupted coupling between GPR39 and TMEM16A in the intestinal FLCs. Meanwhile, over-expression of GPR39 contributes to development of esophageal squamous cell carcinoma [24]; while TMEM16A also serves as a marker for gastrointestinal stromal tumors (GIST) [25]. Although GISTs are thought to originate from ICCs, a subset of GIST display a similar expression pattern as observed in cultured FLCs (TMEM16A, CD34 and PDGFRα positive, but c-Kit negative) [25], [26]. Therefore whether these tumors are originated from FLCs, and whether coupling between GPR39 and TMEM16A plays a role in GI tumorigenesis may be worth further investigation.

In conclusion, our data shows that GPR39 is highly expressed in intestinal FLCs isolated from the muscularis externa. Its activation leads to the opening of TMEM16A channels and depolarization of these cells. The coupling mechanism between GPR39 and TMEM16A in the intestinal FLCs might provide a framework for further studies in GPR39 biology.

## Materials and Methods

### Isolation and Culture of Fibroblast-like Cells

Intestinal fibroblast-like cells were isolated from wild-type C57BL/6 mice (Charles River) or *Gpr39* ^−/−^ mice (Deltagen). All the procedures were performed according to UK Home Office regulations. Briefly, animals (8∼12 weeks) were deeply anesthetized before being sacrificed. The small intestines were removed and the muscularis externa stripped. The tissue was cut into pieces 5–6 mm in length, and digested at 37°C for 2 hours in Ca^2+^/Mg^2+^-free Dulbecco's Phosphate-Buffered Saline (Invitrogen) containing 1.25 mg/ml collagenase, type IV and 0.125 mg/ml DNase (Worthington Enzymes). After the incubation, tissues were dissociated into single cells by passing through fire-polished glass pasteur pipettes. Aliquots were placed onto glass coverslips and cultured in a humidified atmosphere containing 5% CO_2_ at 37°C. Cells were cultured in Minimum Essential Medium (Invitrogen), supplemented with 10% fetal calf serum, antibiotics and antimycotics. Culture medium was changed every 2–3 days.

### Immunocytochemistry

Cells were stained according to standard protocols. Briefly, cells were washed 3 times in PBS prior to being fixed in ice-cold 4% paraformaldehyde for 15 mins. The cells were washed a further 3 times in PBS and then blocked in PBS containing 5% donkey serum, 1% BSA and 0.3% triton X100. The cells were then incubated overnight at 4°C in PBS containing 1% BSA and 1∶100 dilution of primary antibodies: anti-CD34 (BD Biosciences), anti-SK3 (Alomone) or anti-PDGFRα (Abcam). Cells were then rinsed 3 times in PBS before being incubated in a FITC or Cy3-conjugated secondary antibody for 3 hours at 4°C. Finally, the cells were rinsed a further 3 times in PBS before being mounted in Vectashield containing DAPI (Vector).

### PCR

RNA was extracted from cultured enteric neurons and animal tissues using an RNeasy mini kit (Qiagen) according to the manufacturer’s recommended protocol. The samples were tested for concentration/RNA integrity using an Agilent 2100 Bioanalyser. 1 µg of total RNA from each sample was reverse transcribed using SuperScript™ III reverse transcriptase and a random hexamer primer (Invitrogen) according to the manufacturer’s instructions. All reagents used for real-time PCR were purchased from Applied Biosystems and were used according to the manufacturer’s instructions. Individual PCR reactions were set up with 20 ng of cDNA and samples were analyzed on an Applied Biosystems 7500Fast system. Samples were run as uniplex in triplicate using the following gene-specific probes: *Gpr39*: Mm01308380_s1; *Tmem16a*: Mm0072442424_m1 (Applied Biosystems).

In Figure 5E, *Tmem16a* was detected by 30 cycles of amplification with primer sets AATCGCGCACGAGGCACAG and AAGCGTTTGTCATCTTCATGGTAATCC. The expected size of PCR product is 459 base pair.

### Calcium Imaging

Cells grown on 28 mm glass coverslips were loaded with Fura-2AM (2 µM) (Invitrogen) at 37°C for 1 hour in HEPES Buffer Saline Solution (HBSS) containing (in mM): 140 NaCl, 5 KCl, 10 Hepes, 10 Glucose, 1 MgCl_2_, 2 CaCl_2_, pH7.4. Images were acquired every 2 seconds through a 40X oil immersion objective of an Olympus IX81 microscope, using CellR software and a CCD Hamamatsu ORCA-ER camera. Fluorescence intensity was expressed as the ratio of fluorescence at 340 and 380 nm (R340/380).

### Electrophysiology

All the experiments were performed at room temperature. Cells were superfused with HBSS, except NMDG-Cl experiments. Patch electrodes had resistances of 2–4 MΩ, and were filled with solution containing (in mM): 145 KCl, 10 HEPES, 1 MgCl_2_, pH7.3; or 150 CsCl, 10 HEPES, pH7.3 in some experiments as indicated. Electrical signals were recorded by a MultiClamp 700B amplifier, a Digidata 1322A converter and pCLAMP 10 software (Axon).

In voltage clamp configuration, currents were measured by ramping from −100 mV to +100 mV (applied at 0.1 Hz over 200 ms period) from a holding potential of −80 mV. Perforation was achieved with either 10 µg/ml gramicidin or 60 µg/ml of amphotericin-B. No difference was observed between the currents recorded with these two reagents. For Fig. 4A, membrane potentials were continuously recorded in I = 0 mode (zero current clamp).

For whole-cell recordings performed with symmetrical NMDG-Cl solutions, cells were superfused with solution containing (in mM): 140 NMDG, 10 HEPES, 2 CaCl_2_, 1 MgCl_2_, pH to 7.4 with HCl. Pipette solution contains: 145 NMDG, 10 HEPES, pH 7.3 with HCl.

### *Tmem16a* Silencing using siRNA

For gene silencing experiments, cells were reverse transfected with either *Tmem16a* siRNA (s97830, Applied Biosystems) or dH_2_O (mock transfection) using RNAiMAX (Invitrogen) as described by the manufacturer. The final concentration of siRNA in the medium was 20 nM. 72 h after transfection, cells were subjected to gene expression analysis or functional analysis.

### Statistical Analysis

All statistical analyses were conducted using the Origin 7.5 software (OriginLab). Data were expressed as the mean±SEM from n ≥ 3 separate experiments, and subjected to two-sample *t *test. A *p* value of *p*≤0.05 was considered statistically significant with * indicating *p*<0.05, ** *p*<0.01 and *** *p*<0.001.

## Supporting Information

Figure S1 Cultured myenteric neurons [27] were loaded with Fura2-AM for 1 hour at 37°C. Cells were then challenged with 100 µM Zn^2+^. No apparent Ca^2+^ signal was observed. The neuronal status of the cells was confirmed by depolarizing the cells with 75 mM K^+^ at the end of each experiment.
(TIF)

Figure S2 A field with mixed cell populations was chosen for the illustration purpose. **(A)** A bright field image: 3 cobblestone-like cells were marked with green arrowheads, while 4 cells with different morphology labeled with red arrowheads. **(B)** Corresponding Ca^2+^ signals were recorded from the labeled cells. **(C)** Ratiometric images of Ca^2+^ signals at the basal level, and after 100 µM Zn^2+^/100 µM ATP challenge.
(TIF)

Figure S3 Cultured FLCs are stained positively for SK3 antibody.
(TIF)

Figure S4 7 days after culturing, FLCs were used to construct the dose response curve for the Zn^2+^ induced Ca^2+^ signals. EC_50_ = 30.4 µM (Ca^2+^ imaging).
(TIF)

Figure S5 While Zn^2+^ has an evident inhibitory effect on the basal outward current, it did not induce any current in FLCs if the cells were pre-treated with 30 µM CaCC_inh_-A01. Two minutes after Zn^2+^ application, the outward currents are 1636±214.3 pA in controls, and −81.4±82.1 pA in CaCCinh-A01 treated cells (p<0.001, n = 7). A representative trace is shown for the time course of the current amplitude recorded at −80 and +80 mV.
(TIF)
