# Supplementary figures and images for: GPR39 Is Coupled to TMEM16A in Intestinal Fibroblast-Like Cells
Source: PLoS One. 2012 Oct 25;7(10):e47686. doi: 10.1371/journal.pone.0047686 (PMC3485058; doi:10.1371/journal.pone.0047686)

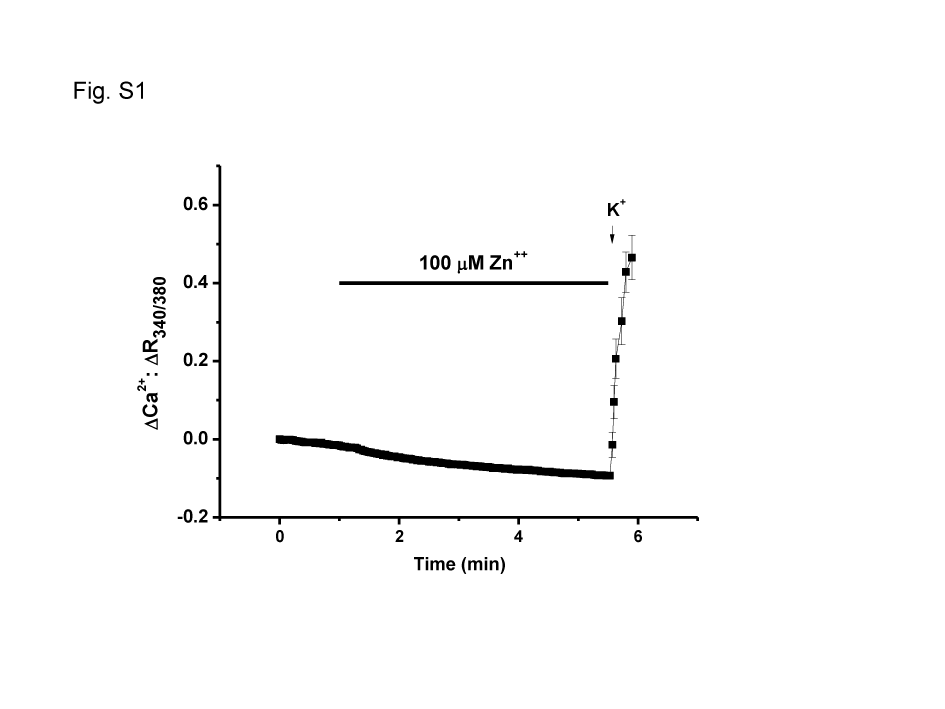

Supplement: Figure S1 — Cultured myenteric neurons [27] were loaded with Fura2-AM for 1 hour at 37°C. Cells were then challenged with 100 µM Zn2+. No apparent Ca2+ signal was observed. The neuronal status of the cells was confirmed by depolarizing the cells with 75 mM K+ at the end of each experiment. (TIF) [file pone.0047686.s001.tif]

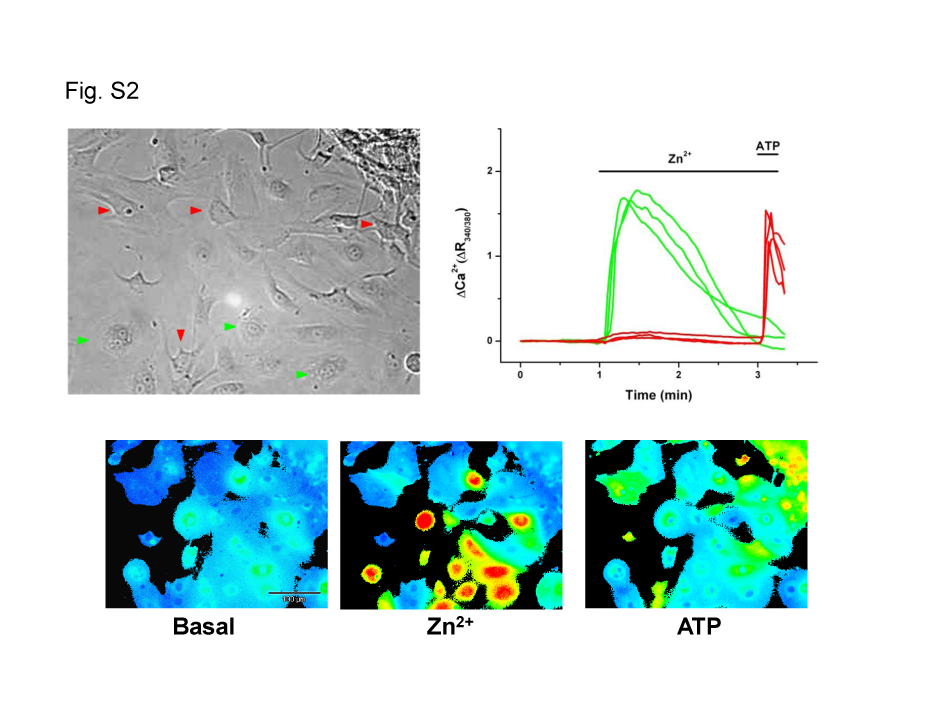

Supplement: Figure S2 — A field with mixed cell populations was chosen for the illustration purpose. (A) A bright field image: 3 cobblestone-like cells were marked with green arrowheads, while 4 cells with different morphology labeled with red arrowheads. (B) Corresponding Ca2+ signals were recorded from the labeled cells. (C) Ratiometric images of Ca2+ signals at the basal level, and after 100 µM Zn2+/100 µM ATP challenge. (TIF) [file pone.0047686.s002.tif]

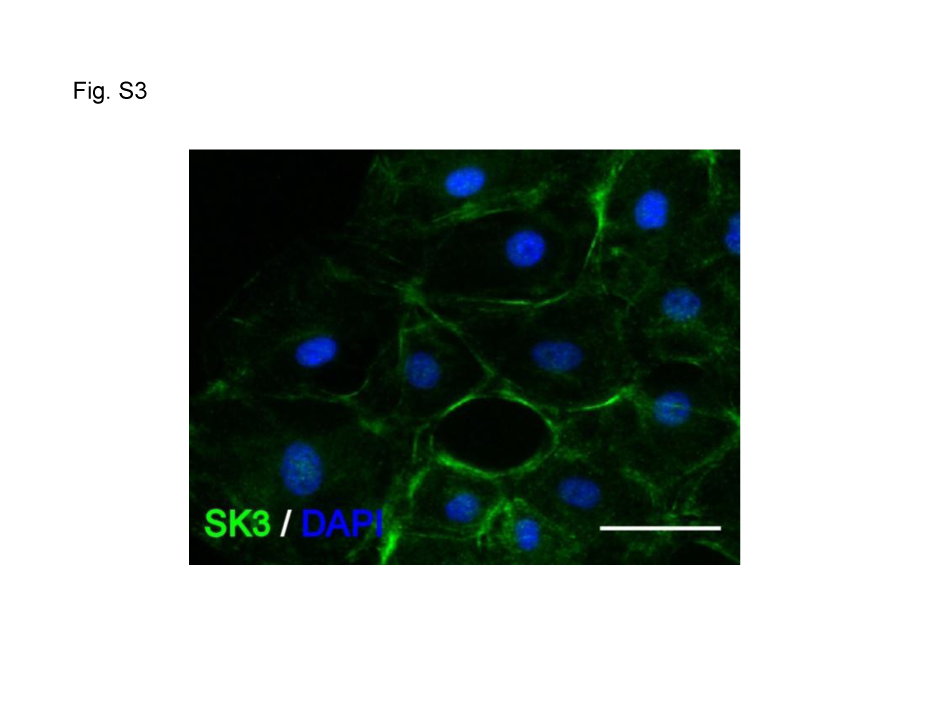

Supplement: Figure S3 — Cultured FLCs are stained positively for SK3 antibody. (TIF) [file pone.0047686.s003.tif]

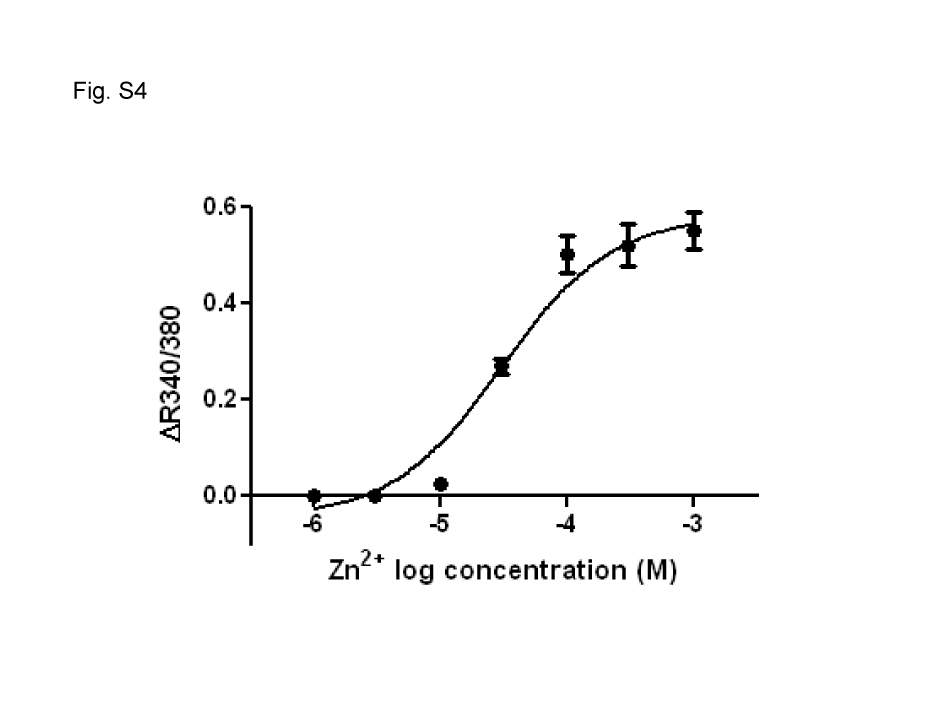

Supplement: Figure S4 — 7 days after culturing, FLCs were used to construct the dose response curve for the Zn2+ induced Ca2+ signals. EC50 = 30.4 µM (Ca2+ imaging). (TIF) [file pone.0047686.s004.tif]

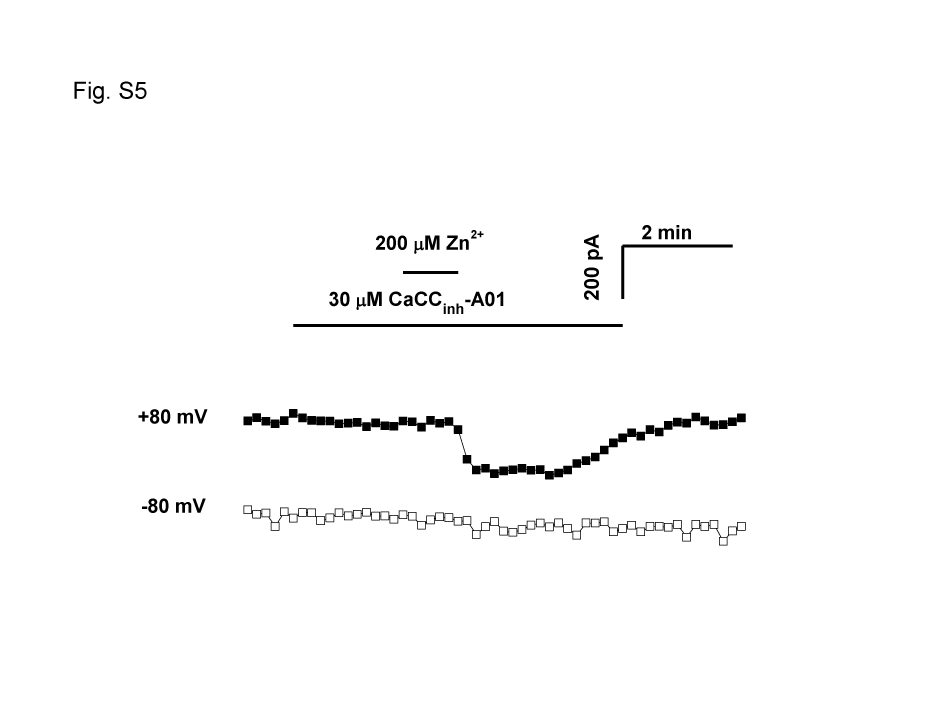

Supplement: Figure S5 — While Zn2+ has an evident inhibitory effect on the basal outward current, it did not induce any current in FLCs if the cells were pre-treated with 30 µM CaCCinh-A01. Two minutes after Zn2+ application, the outward currents are 1636±214.3 pA in controls, and −81.4±82.1 pA in CaCCinh-A01 treated cells (p<0.001, n = 7). A representative trace is shown for the time course of the current amplitude recorded at −80 and +80 mV. (TIF) [file pone.0047686.s005.tif]
